# Use of Intrathecal and Intravenous Clonidine in A Case of Severe Tetanus with Acute Renal Failure

**Published:** 2009-04

**Authors:** Alok Kumar, Raktima Anand, Anita Rahal, Sandhya Od

**Affiliations:** 1 Senior resident, Department of Anaesthesiology and Intensive Care Maulana Azad Medical College & Associated Lok Nayak Hospital, New Delhi- 110002, 3. B.S.A. Hospital, Delhi; 2 Director, Professor and Head, Department of Anaesthesiology and Intensive Care Maulana Azad Medical College & Associated Lok Nayak Hospital, New Delhi- 110002, 3. B.S.A. Hospital, Delhi; 3 Medical Officer, Department of Anaesthesiology and Intensive Care Maulana Azad Medical College & Associated Lok Nayak Hospital, New Delhi- 110002, 3. B.S.A. Hospital, Delhi; 4 Senior resident, Department of Anaesthesiology and Intensive Care Maulana Azad Medical College & Associated Lok Nayak Hospital, New Delhi- 110002, 3. B.S.A. Hospital, Delhi

**Keywords:** Tetanus, Clonidine, Intrathecal

## Abstract

**Summary:**

Tetanus is an acute often fatal disease produced by the Gram-positive, obligate anaerobic bacterium Clostridium tetani. Prolonged intensive care is required in severe tetanus, with the associated complications including nosocomial sepsis. Autonomic dysfunction in severe tetanus is difficult to manage and is a significant cause of mortality. We present here, use of clonidine in a case of severe tetanus with acute renal failure who was successfully managed.

## Introduction

Tetanus is caused by the exotoxins produced by Clostridium tetani. The disease is characterised by generalised muscle rigidity, autonomic instability and sometimes convulsions^1^. Autonomic dysfunction in severe tetanus is difficult to control and is a significant cause of mortality for which treatment includes the use of heavy sedation, peripheral adrenergic blocking agents^2^ chlorpromazine^2^, ganglion-blockers^2^, morphine^3^ magnesium sulphate^4^ clonidine^4^ baclofen^5^ and atropine^6^ often in combination. Other strategies include epidural analgesia^7^ and atrial pacing^8^. This case report emphasises the role of intrathecal and intravenous clonidine in a tetanus patient with acute renal failure and provides a novel way to successfully manage these patients.

## Case report

An 18-year-old male patient weighing 58 kg was admitted to medicine ward with complaints of acute onset of muscular pain one day back with stiffness in body and fever (102°F) for which he had taken some analgesics from a private practitioner. He gave past history of superficial open wound on right elbow one month back which had healed. He had received Inj. Tetanus toxoid (TT) at that time. Tetanus was suspected along with a differential diagnosis of encephalitis and meningitis. He was admitted and given Inj. TT, antitetanus serum 1500 I.U., and metronidazlole. However patient had muscle spasms and complained of difficulty in breathing following which he was intubated and shifted to intensive care unit. Diazepam 10 mg.hour^−1^, rocuronium 30 mg.hour^−1^ by infusion and methocarbamol 100 mg TDS, chlorpromazine 50 mg BD were started. SIMV(Pressure control) with pressure support mode of ventilation with pressure control 20cmH_2_O, pressure support 20cmH_2_O upper pressure limit 40cmH_2_O, SIMV rate 12/minute, FiO_2_ 0.4 with appropriate minute ventilation alarm limits on Siemens Servo ventilator 300 was used for ventilation.

Apart from the routine investigations lumbar puncture for CSF examination, fundal examination and CT scan head were performed. CSF examination revealed increase in proteins.

Patient required increasing doses of rocuronium for effective ventilation as was assessed from increase in inspiratory pressures and low minute ventilation alarms. On the second day, patient developed autonomic instability with wide fluctuations in heart rate and blood pressure (heart rate ranged from 40 to 130/minute, systolic blood pressure from 65 to 180mmHg and diastolic blood pressure from 40 to110 mmHg). These fluctuations were marked at the time of endotracheal suctioning and patient turning. Infusion of magnesium sulphate at 2 gm.hour^−1^ was added after a loading dose of 4 gm for adequate control of spasms and prevention of autonomic instability.

On the fourth day, patient developed fever (102°F), decreased urine output (300 ml), elevated blood urea nitrogen (38mg/dl) and serum creatinine (2.4mg/dl) levels. The serum magnesium levels were 7.0 mEq/L. Meropenem 500 mg i.v. TDS was started after sending culture sensitivity reports based on suspicion of sepsis and culture sensitivity profile prevalent in our ICU. Cental venous pressure (using triple lumen CVP catheter 16G lumen size in right internal jugular vein) was within normal limits. Magnesium was stopped. Atracurium was used for paralysis, rocuronium was stopped. However patient had spasms and fluctuations in heart rate and blood pressure. On the 5^th^ day epidural catheter (Portex, 20G) was inserted in subarachnoid space using 18G Tuohy's needle with patient in lateral decubitus position and threaded 3 cm cephalic. We administered clonidine 15 μg intrathecally every 4 hourly through the catheter along with intravenous infusion of clonidine 50 μg/hour. Clonidine which is available as 150 μg/ml was diluted in normal saline to concentration of 15 μg/ml and injected intrathecally after taking in consideration the dead space of the catheter (about 0.3ml).

Intravenous fluids (Ringer's lactate and dextrose 5% in normal saline) were administered to keep CVP in normal range. Strict intake and output charting was maintained. Patient was tracheostomised. Kidney function tests and serum magnesium levels were done daily. Patient was hemodynamically stable (heart rate ranged from 76 to 110/minute, systolic blood pressure from 106 to 130 mmHg and diastolic blood pressure from 75 to 90 mmHg, after about 24 hours of starting clonidine. Kidney functions improved (blood urea nitrogen 22mg/dl on 7^th^ day and 17mg/dl on 10^th^ day and serum creatinine 1.8mg/dl on 7^th^ day and 1.3mg/dl on 10^th^ day) and magnesium levels decreased. On the seventh day atracurium was stopped. Patient thereafter had no spasms. Intrathecal clonidine was reduced to 15 μg every 8 hourly from 9^th^ day. Diazepam and methocarbazole were stopped and intrathecal catheter removed on the 11^th^ day. Patient was maintained on i.v. clonidine at 25 μg/hour from 11^th^ day which was stopped on the 13^th^ day. Active physiotherapy was started and patient was weaned off the ventilator on 17^th^ day. Tracheostomy was closed subsequently and patient discharged on 25^th^ day.

## Discussion

Tetanus is caused by the Gram-positive bacillus, Clostridium tetani. It is a spore forming obligate anaerobe normally found in soil. It is rarely cultured from wounds and the diagnosis is a clinical one. It produces two exotoxins, tetanospasmin and tetanolysin. Tetanolysin damages local tissue and provides optimal conditions for bacterial multiplication. It is therefore important to perform a wide debridement of any wound suspected of being a portal of entry for the bacteria. Tetanospasmin binds irreversibly preventing inhibitory neurotransmitter release. The incubation period is 7-10 days and spasms commonly occur 1-7 days later. The classical clinical triad of rigidity, muscle spasms and autonomic dysfunction is usually seen in severe tetanus^1^.

There are no laboratory findings characteristic of tetanus. The diagnosis is entirely clinical and does not depend upon bacteriologic confirmation. However CSF examination may reveal raised proteins and immunoglobulins (IgG) in patients with severe disease^9^.

The severe muscle spasms and autonomic instability affect the respiratory and cardiovascular systems, generally requiring treatment in the intensive care unit (ICU). The proposed mechanisms for autonomic dysfunction include: - the effect of toxin on the brainstem and autonomic interneurones causing impairment of inhibitory pathways (the most likely mechanism), a direct effect of toxin on the myocardium, loss of inhibition of the adrenal medulla with increased adrenaline secretion, direct inhibition of the release of endogenous opiates by tetanospasmin, increased release of thyroid hormone^10^,^11^.

Sympathetic over activity is the major cause of tetanus-related complications in the intensive care unit. Treatment is directed at suppressing rigidity, muscle spasms, and sympathetic activation, and controlling autonomic instability. Methocarbamol is a central muscle relaxant for skeletal muscles, used to treat spasms. It is structurally related to guaifenesin. Methocarbamol's exact mechanism of causing skeletal muscle relaxation is unknown. It is thought to work centrally, perhaps by general depressant effects. It has no direct relaxant effects on striated muscle, nerve fibers, or the motor endplate. It will not directly relax contracted skeletal muscles rather produces a depression of polysynaptic pathways^12^. The drug has a secondary sedative effect. Diazepam has long been reported to be effective in the treatment of tetanus due to the combined anticonvulsant and muscle relaxation actions on tetanus muscle spasms and rigidity. Also, it has sedative and anxiolytic effects. Chlorpromazine also has anticonvulsant effect and muscle relaxant properties. Magnesium sulphate has been used both in artificially ventilated patients to reduce autonomic disturbance and in non-ventilated patients to control spasms. Magnesium is a pre-synaptic neuromuscular blocker, blocks catecholamine release from nerves and adrenal medulla, reduces receptor responsiveness to released catecholamines, is an anticonvulsant and a vasodilator^4^.

Renal failure in severe tetanus is not uncommon and can occur secondary to autonomic instability, sepsis, myogloniuria with hypotension or aciduria^13^.

Sepsis is an important consideration in these patients as these patients need ventilatory support for weeks. It can lead to acute renal failure. Magnesium sulphate when given in cases of renal failure will cause toxic levels of magnesium leading to numerous cardiovascular complications, besides affecting the respiratory, nervous and coagulation systems.

As the patient developed acute renal failure and also required increasing doses of rocuronium for effective ventilation, we preferred to use atracurium instead of rocuronium. It has been reported that the elimination half life of rocuronium is increased in renal failure because of an increase in volume of distribution with no change in clearance whereas the pharmacokinetics and duration of action of atracurium are unaffected by renal failure^14^.

The clinical significance is not significant but the availability and the reported use of atracurium infusion in tetanus with no cumulation of laudanosine, the epileptogenic metabolite of atracurium prompted its use inthis patient with acute renal failure^15^,^16^. However rocuronium which does not have active metabolites can be continued in similar patients as prompt reversal of neuromuscular blockade is not required.

Clonidine is a selective partial agonist for alpha-2 adrenergic receptors with a 200-fold selectivity for the alpha-2-receptor over the alpha-1. Alpha-2 agonists have both peripheral and central effects on the cardiovascular system. Acting centrally, clonidine causes hypotension and bradycardia. The precise mechanism of action is not fully understood, but is thought to involve inhibition of sympathetic outflow and potentiation of parasympathetic nervous activity, increasing vagal tone. Acting peripherally, clonidine inhibits the release of noradrenaline from prejunctional nerve endings. It also produces marked sedation and anxiolysis, decreases spontaneous motor activity and potentiates the sedative and anaesthetic actions of other drugs. Two case studies report the use of clonidine in the management of autonomic dysfunction in tetanus with conflicting results^17^,^18^. It has been reported that no appreciable difference in catecholamine levels occur following administration of alpha-2 agonists in the doses clinically used, although clonidine did allow easier control of autonomic crises than with other agents^19^.

The excretion of clonidine is not dependent on renal function. When we administered clonidine intrathecally, as well as intravenously, it allowed us to omit magnesium sulphate, decrease the infusion rate of diazepam, besides excellent control of seizures and provided autonomic stability. Clonidine is known to cause hypotension and bradycardia and therefore would have limited its use in our patient who had sepsis when used in higher doses. Intrathecal administration allowed us to control spasms better and provide stable haemodynamics, and reduced the intravenous requirements of clonidine and other drugs used for control of spasms and sedation.

Rebound hypertensive episodes have been reported after withdrawal of long-term clonidine treatment, although short-term use of clonidine proved to be safe^20^. The clonidine dose should be decreased slowly so as to avoid rebound hypertension.

Clonidine after renal ischemia has been shown to lessen acute renal failure and microvascular damage. These results suggest that clonidine may be beneficial because it prevents ischemic microvascular injury in the renal outer medulla, an effect that may decrease tubular obstruction by lessening desquamation of damaged tubular cells or cell constituents into the tubular lumen. Clonidine may also decrease formation of obstructive hyaline casts in collecting ducts by blunting the kidney's response to vasopressin and increasing tubular fluid flow rate^21^.

In conclusion this case report suggests that clonidine used intrathecally as well as intravenously is beneficial in management of tetanus and has the advantages of controlling spasms, sympathetic over activity, provide stable haemodynamics, reduce the intravenous requirements of clonidine and other drugs used for control of spasms and sedation with beneficial effects in renal failure.
